# Low Serum miR-607 Level as a Potential Diagnostic and Prognostic Biomarker in Patients of Pancreatic Ductal Adenocarcinoma: A Preliminary Study

**DOI:** 10.1155/2021/8882129

**Published:** 2021-06-11

**Authors:** Dawei Jiang, Xiangfei Yuan, Jianqi Ni, Lan Shen, Min Cai, Liu Xu

**Affiliations:** ^1^Department of Hepatobiliary Surgery, The First Hospital of Jiaxing, Affiliated Hospital of Jiaxing University, Jiaxing 314001, China; ^2^Tianjin Institute of Integrative Medicine for Acute Abdominal Diseases, Tianjin Nankai Hospital, Tianjin 300100, China

## Abstract

**Background:**

One of the microRNAs (miRNAs) known to be associated with cancer development is miR-607. The aim of this study is to investigate the clinical significance and diagnostic and prognostic value of miR-607 and to explore its potential role in pancreatic ductal adenocarcinoma (PDAC).

**Methods:**

The expression levels of miR-607 were assessed by quantitative real-time polymerase chain reaction (qRT-PCR). The correlation between miR-607 expression and clinical characteristics was analyzed by the Chi-square test. Overall survival (OS) and progression-free survival (PFS) were evaluated via the Kaplan–Meier method, and the association between miR-607 expression and OS was investigated by the Cox proportional hazard analysis. The diagnostic value was estimated via receiver operating characteristic (ROC) curve analysis. The effect of miR-607 overexpression on cell migration, invasion, and epithelial-mesenchymal transition (EMT) was determined by wound-healing, Transwell invasion, and Western blotting assays.

**Results:**

miR-607 levels were downregulated in PDAC tumor tissues compared with normal tissues. Also, low miR-607 levels were observed in serum samples from PDAC patients than that in healthy controls. The miR-607 level was found to be closely correlated with lymphatic metastasis and liver metastasis, perineural invasion, and OS and PFS, and the low miR-607 level was an independent prognostic factor for the poor OS of PDAC patients. Furthermore, the area under the curve (AUC) of serum miR-607 for discriminating PDAC patients was 0.785 with a sensitivity of 0.647 and a specificity of 0.772, which was better than those for CA19-9 (AUC: 0.702, sensitivity: 0.607, specificity: 0.736) and CEA (AUC: 0.648, sensitivity: 0.542, specificity: 0.670). The AUC (0.863), sensitivity (0.766), and specificity (0.831) of their combination in the diagnosis of PDAC were better than those for alone. Moreover, ectopic overexpression of miR-607 could inhibit cell migration and invasion of BxPc-3 and PANC-1 cells by decreasing EMT ability.

**Conclusions:**

Low serum miR-607 level may serve as a potential diagnostic and prognostic biomarker through regulation of tumor metastasis in PDAC patients.

## 1. Introduction

Pancreatic cancer is the fourth leading cause of cancer-related mortality in the USA and is characterized by malignant phenotypes with local invasion, distant metastasis, and resistance to chemotherapy and radiation therapy [1, 2]. Almost 95% of histological types for pancreatic cancer are pancreatic ductal adenocarcinomas (PDACs), which result in poor prognosis than other types of pancreatic cancer [3]. Patients with PDAC were usually diagnosed at later stages due to insidious early symptoms, and the five-year relative survival rate is only 3%–5% [4]. Currently, the diagnostic approaches for PDAC mainly rely on surgical pathological biopsy, which is an invasive method and causes greater pain to patients [5, 6]. Therefore, study on the pathogenesis of PDAC at the molecular level is highly needed for developing new diagnostic biomarkers and effective therapeutic strategies and improving the prognosis of PDAC.

MicroRNAs (miRNAs) are a group of endogenous, small, noncoding RNA consisting of 20–24 nt, which are posttranscriptional regulators that inhibit the expression of one or more target genes via binding with 3′-untranslated regions (UTRs) of mRNAs [7]. Numerous studies have demonstrated that dysregulation of miRNAs is associated with the tumorigenesis and development of various types of human cancer [8, 9]. For instance, Leone et al. [10] summarized the function of various miRNAs and their targets in tumor angiogenesis and described the strategies and challenges of miRNA-based antiangiogenic therapies. In prostate cancer, Liu et al. reported [11] that miR-21 induces angiogenesis through AKT and ERK activation and HIF-1*α* expression. Also, Wang et al. [12] found that let-7a regulates angiogenesis through posttranscriptional regulation of TGFBR3. Therefore, miRNAs represent a novel therapeutic window for cancer by targeting both the tumor and the microenvironment.

Increasing researches have shown that tumor-specific circulating miRNAs can stably exist in various body fluids of human including serum, urine, gastric juice, synovial fluid, and amniotic fluid and serve as novel, noninvasive, optimized biomarkers for the diagnosis of various malignancies including PDAC [13]. For example, Fendereski et al. [14] demonstrated that miR-196a is a potential diagnostic biomarker for esophageal squamous cell carcinoma. Park et al. [15] showed that miR-944 overexpression is a biomarker for poor prognosis of advanced cervical cancer. Li et al. [16] reported that serum miR-381 is a potential marker for early diagnosis of gastric cancer. In addition, Kawamura et al. [17] revealed that miR-4525, miR-451a, and miR-21 in portal vein blood are high-sensitive liquid biomarkers for the selection of high-risk PDAC patients.

Since its report on quelling the Ebola virus by in silico approach, miR-607 has been linked to biological behaviors of different human diseases [18]. In chronic lymphocytic leukemia, miR-607 is expressed lower in tumor tissues and suppresses cell proliferation, arrests cell cycle progression, and induces cellular apoptosis [19]. In cervical cancer, miR-607 expression is downregulated in cervical cancer tissues and decreases cell proliferation, migration, and invasion abilities [20]. To our knowledge, there are no other further studies on the clinical significance and role of miR-607 in PDAC. In this study, the prognostic and diagnostic value of miR-607 was investigated in 50 pairs of PDAC tumor tissues and adjacent normal tissues and 184 pairs of serum samples from PDAC patients and healthy controls. And the association between miR-607 expression and clinical characteristics of PDAC patients was analyzed. Furthermore, the effects of miR-607 overexpression on cell migration, invasion, and epithelial-mesenchymal transition (EMT) were explored. Our results suggested that low serum miR-607 level may represent a novel indicator of diagnosis and prognosis in patients with PDAC.

## 2. Materials and Methods

### 2.1. Study Population

A total of 50 pairs of PDAC tumor tissues and adjacent normal tissues were collected from the Department of Hepatobiliary Surgery, Affiliated Hospital of Jiaxing University, between March 2016 and March 2019, and the cases' data on age, gender, tumor differentiation, tumor size, T stage, clinical stage, perineural invasion, lymphatic metastasis, liver metastasis, CA199 level, and CEA level were retrospectively obtained from medical records. The patients' population included 31 men and 19 women with an average age of 56.12 ± 6.24 years (range: 20 to 83 years). Moreover, a large set of serum samples were collected from 184 PDAC patients (96 males and 88 females; the average age of 52.74 ± 8.05 years; range from 26 to 80 years) and from 184 healthy people (110 males and 74 females; the average age of 50.95 ± 7.39 years; range from 19 to 71 years) as control. All patients have not received any preoperative antineoplastic therapy. All samples were immediately frozen in liquid nitrogen and stored at −80°C until RNA extraction. The research was approved by the Ethics Committees at Affiliated Hospital of Jiaxing University in accordance with the regulations of the Declaration of Helsinki, and consent forms were signed by all patients. The 5-year overall survival (OS) and progression-free survival (PFS) of PDAC patients after surgery were obtained from follow-up telephone.

### 2.2. Cell Lines

PDAC cell lines BxPc-3 (CRL-1687™) and PANC-1 (CRL-1469™) were purchased from the American Type Culture Collection (ATCC, Manassas, VA, USA) and were maintained in DMEM medium (Invitrogen, Carlsbad, CA, USA) containing 10% FBS and 1% antibiotics. The immortalized pancreatic ductal epithelial cell line HPDE6c7 (CVCL-0P38™) was obtained from the Chinese Academy of Sciences (Shanghai, China) and was incubated in Keratinocyte Serum-Free Growth Medium (Invitrogen) containing 1% antibiotics and 0.2 ng/ml recombinant endothelial growth factor (Sigma, St Louis, MO, USA). All cells were grown in a humidified incubator at 37°C with 5% CO_2_.

### 2.3. RNA Isolation and Quantitative Real-Time Polymerase Chain Reaction (qRT-PCR)

The total RNA from human PDAC cells and tissues was extracted by TRIzol reagents (Invitrogen), according to the manufacturer's protocol. 1 *μ*g of total RNA was reverse transcribed to cDNA, and the cDNA was used for determining the expression levels of miR-607 by a miScript SYBR^®^ Green PCR kit (Qiagen, Germany) with the specific primers. All reactions were run on Applied Biosystems 7500 Real-Time PCR system (Applied Biosystems, Foster City, CA, USA), according to the instruction. U6 was served as an internal normalized reference. The specific primers used for qRT-PCR were as follows: miR-607, 5′-CAGGCATCGTTCAAATCC-3′ (sense), universal primer (antisense); U6: 5′-CTCGCTTCGGCAGCACA-3′ (sense), 5′-AACGCTTCACGAATTTGCGT-3′ (antisense). Relative expression of miR-607 was analyzed by the comparative threshold cycle (Ct) (2^−ΔΔCt^) method [21].

### 2.4. miRNAs Transfection

The miR-607 mimics and mimics control were synthesized and purified by GenePharma (Shanghai, China). For miRNA transfection, PDAC cells were cultured in 6-well cell plates overnight, and then 50 nmol/L of miR-607 mimics and mimics control were performed into each well by Lipofectamine 2000 reagents (Invitrogen), according to the manufacturer's protocol. After incubation for 48 h at 37°C with 5% CO_2_, qRT-PCR was conducted to detect the expression levels of miR-607.

### 2.5. Western Blotting Analysis

Total protein from human PDAC cells was extracted by protein lysis buffer (Invitrogen) containing 2 *μ*L protease inhibitor, according to the instruction. Protein concentration was determined by a BCA Protein assay kit (Millipore, Darmstadt, Germany). 30 mg of protein in each sample was separated by polyacrylamide gel electrophoresis via 10% separating gel and transferred to 0.22 *µ*m PVDF membranes (Millipore). Then, membranes were blocked with 5% nonfat milk for 2 h at 37°C and followed incubated with anti-MMP-2 (no. sc-13594; 1 : 1000; Santa Cruz Biotechnology, Santa Cruz, CA, USA), anti-MMP-9 (no. sc-393859; 1 : 750; Santa Cruz Biotechnology), anti-N-cadherin (no. sc-59987; 1 : 1500; Santa Cruz Biotechnology), anti-E-cadherin (no. sc-8426; 1 : 2000; Santa Cruz Biotechnology), anti-Vimentin (no. sc-6260; 1 : 1000; Santa Cruz Biotechnology), anti-Snail (no. sc-271977; 1 : 500; Santa Cruz Biotechnology), and GAPDH (no. sc-47724; 1 : 5000; Santa Cruz Biotechnology) antibodies overnight at 4°C. Goat anti-mouse horseradish peroxidase-conjugated IgG (no. sc-2005; 1 : 2500; Santa Cruz Biotechnology) was used as the secondary antibody and incubated with the membranes for 1 h at 37°C. Finally, protein bands were observed using the enhanced chemiluminescence kit (Millipore) on Chemidoc XRS Gel Imaging System (Bio-Rad, Hercules, CA, USA).

### 2.6. Transwell Invasion Assay

The invasion of PDAC cells was determined by a Transwell chamber coated Matrigel (8 *µ*m pore size; BD Biosciences, San Jose, CA, USA). Briefly, cells were transfected with miR-607 mimics and mimics control for 24 h, and 5 × 10^4^ cells were collected and then transferred to the top of chambers in a serum-free 200 *μ*L DMEM medium. 500 *μ*L DMEM containing 15% FBS was added to the lower chamber. After incubation for 24 h, the noninvading cells were removed by a cotton swab, and the invading cells were fixed with 95% ethanol for 30 min at 37°C and stained with 0.1% crystal violet for 15 min at 37 °C. The images of invading cells were observed at ×100 and 200 magnifications under an LSM710 inverted light microscope (Zeiss, Germany).

### 2.7. Wound-Healing Assay

The migration of PDAC cells was investigated by wound-healing assay. In brief, cells were transfected with miR-607 mimics and mimics control for 24 h, 3 × 10^6^ cells were collected and seeded in 6-well plates and cultured overnight, and an artificial wound was created using a 200 *μ*l pipette tube. The wound closures were observed after 24 h and imaged under an LSM710 inverted light microscope. To evaluate cell migration rate, we measured the fraction of cell coverage across the line.

### 2.8. Statistical Analysis

Statistical analyses were performed using SPSS 25.0 software (SPSS, Chicago, IL, USA). All numerical data were presented as the means ± standard deviation (SD) for multiple samples. Student's *t*-test or one-way analysis of variance (ANOVA) followed by the Bonferroni posttest was used (two-tailed) for parametric variables. The Chi-square test was performed for the association between miR-607 level and clinicopathologic factors of PDAC patients. When Gaussian distribution cannot be assumed, a nonparametric test (Mann–Whitney *U* test between 2 groups and Kruskal–Wallis *H* test for ≥3 groups) was performed. Overall survival (OS) and progression-free survival (PFS) curves were plotted by the Kaplan–Meier method and compared by the log-rank test. The significance of different variables with respect to OS was analyzed by Cox proportional hazard analysis. The diagnostic value was estimated via receiver operating characteristic (ROC) curve analysis. *P* < 0.05 was considered significant.

## 3. Results

### 3.1. miR-607 Levels Are Downregulated in PDAC Samples and Cell Lines

**Figure 1:**
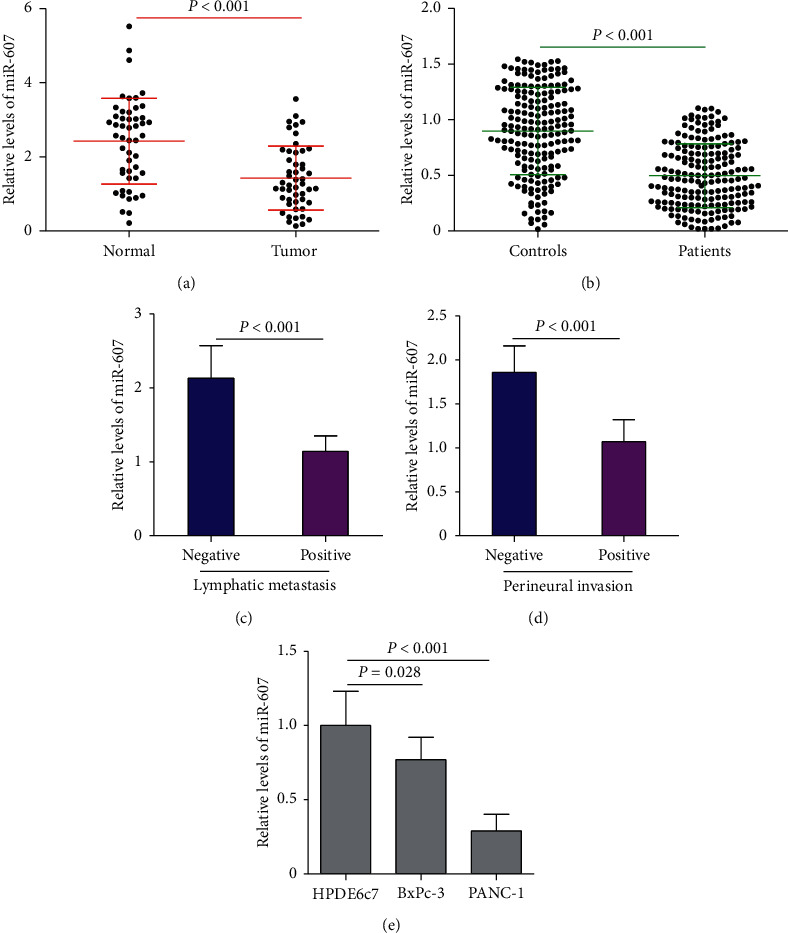
The expression levels of miR-607 in patients with PDAC. (a) qRT-PCR analysis of miR-607 expression levels (normalized to U6) in 50 pairs of PDAC tumor tissues and adjacent normal tissues. (b) Analysis of serum miR-607 levels in 184 pairs of PDAC patients and healthy controls. (c) Comparison of the relative expression levels of miR-607 in PDAC tumor tissues with positive lymphatic metastasis and other PDAC tumor tissues with negative lymphatic metastasis. (d) The expression levels of miR-607 were analyzed in PDAC tumor tissues with positive perineural invasion and other PDAC tumor tissues with negative perineural invasion. (e) Levels of miR-607 in the BxPc-3 and PANC-1 cells are lower than those in the HPDE6c7 cells. qRT-PCR: quantitative real-time polymerase chain reaction, PDAC: pancreatic ductal adenocarcinoma. Data are represented as the mean ± SD from three independent experiments.

To investigate the potential clinical significance of miR-607 in PDAC, we first evaluated miR-607 expression in 50 pairs of PDAC tumor tissues and adjacent normal tissues by qRT-PCR.  Figure 1(a) shows that miR-607 levels were significantly decreased in tumor tissues compared with normal tissues (*P* < 0.001). Also, we assessed the expression of miR-607 in 184 pairs of serum samples from PDAC patients and healthy controls. Results revealed that the serum levels of miR-607 in PDAC patients were significantly lower than those in the healthy controls (Figure 1(b), *P* < 0.001).

Moreover, we found that miR-607 expression in PDAC tumor tissues with positive lymphatic metastasis and perineural invasion was markedly downregulated compared with those tumor tissues with negative lymphatic metastasis and perineural invasion, respectively (Figures 1(c) and 1(d), *P* < 0.001). In addition, miR-607 levels were also compared among HPDE6c7 cells and two PDAC cells (BxPc-3 and PANC-1). Similarly, the expression levels of miR-607 were decreased in the BxPc-3 and PANC-1 cells compared with the HPDE6c7 cells (Figure 1(e), *P* < 0.05).

### 3.2. miR-607 Serves as a Potential Diagnostic Biomarker for Patients with PDAC

**Figure 2:**
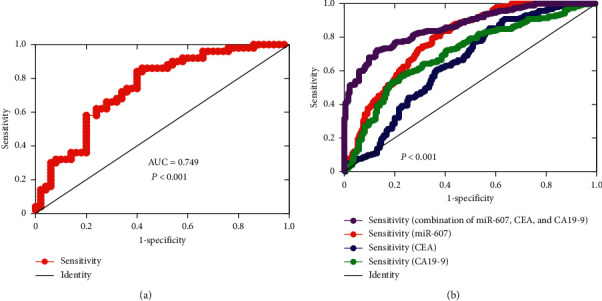
The diagnostic value of miR-607 in patients with PDAC. (a) ROC curve analysis of miR-607 expression for discriminating PDAC tumor tissues from normal tissues. (b) ROC curve analysis based on serum miR-607, CA19-9, CEA, and their combination for differentiating PDAC patients from healthy controls. ROC: receiver operating characteristic, AUC: area under the curve.

In order to evaluate the diagnostic value of miR-607 in PDAC, the expression levels of miR-607 in tumor cancer tissues and normal tissues were plotted as a ROC curve. Results showed a strong separation between the tumor tissues and normal tissues, with an area under the curve (AUC) of 0.749 (95% CI: 0.653–0.845), the sensitivity was 0.639, and the specificity was 0.755 (Figure 2(a), *P* < 0.001). Furthermore, ROC curves of serum miR-607, CA19-9, and CEA levels were investigated in our study. We found that AUC value of miR-607 level (0.785, 95% CI: 0.738–0.831) was larger than AUC values for CA199 level (0.702, 95% CI: 0.649–0.756) and CEA level (0.648, 95% CI: 0.591–0.704) comparing the PDAC patients with the healthy controls (Figure 2(b), *P* < 0.001). Also, the sensitivity and specificity of serum miR-607 level (sensitivity: 0.647, specificity: 0.772) in the diagnosis of PDAC were higher than those for CA19-9 level (sensitivity: 0.607, specificity: 0.736) and CEA level (sensitivity: 0.542, specificity: 0.670). Moreover, the AUC (0.863), sensitivity (0.766), and specificity (0.831) of their combination in the diagnosis of PDAC were better than those for alone (*P* < 0.001).

### 3.3. Low miR-607 Level Is Correlated with Positive Lymphatic Metastasis, Liver Metastasis, Perineural Invasion, and Poor OS and PFS of PDAC Patients

**Figure 3:**
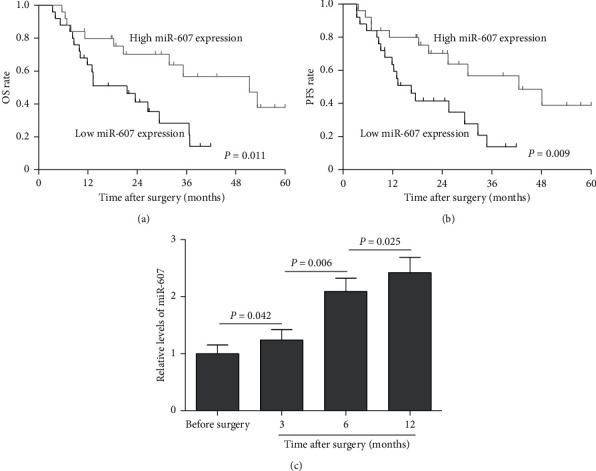
Kaplan–Meier survival curves for patients with PDAC based on miR-607 expression status. (a) Low miR-607 expression group had a significantly shorter OS than high miR-607 expression group. (b) The PFS was significantly worse in PDAC patients with low miR-607 expression than in those patients with high miR-607 expression. (c) qRT-PCR analysis of miR-607 expression levels in PDAC patients before and after surgery. OS: overall survival, PFS: progression-free survival.

To further evaluate the correlation of miR-607 level with clinicopathologic features, the PDAC patients were grouped into two groups including high miR-607 expression group (*n* = 25, miR-607 expression ≥ mean ratio) and low miR-607 expression (*n* = 25, miR-607 expression < median ratio) by using the median miR-607 expression in PDAC tumor tissues as the threshold. A Chi-square test was used to analyze data from 50 cases of PDAC patients. As shown in Table 1, miR-607 expression level showed no association with age, gender, tumor differentiation, tumor size, T stage, clinical stage, CA199 level, and CEA level. However, miR-607 levels showed a significant association with lymphatic metastasis (*P*=0.002), liver metastasis (*P*=0.018), and perineural invasion (*P*=0.004). Furthermore, the log-rank analysis indicated that the OS (*P*=0.011) and PFS (*P*=0.009) were significantly worse in patients with low miR-607 levels than those patients with high miR-607 levels (Figures 3(a) and 3(b), *P* < 0.05).

### 3.4. Low miR-607 Level Is an Independent Prognostic Factor for Poor OS of PDAC Patients

The expression of serum miR-607 was also analyzed in PDAC patients before and after surgery. Interesting, data indicated that miR-607 expression levels were upregulated in PDAC patients after surgery than those before surgery, and its expression level showed an upward trend at 3, 6, and 12 months after surgery, with the highest expression level at 12 months (Figure 3(c), *P* < 0.05). We further used Cox proportional hazard analysis to investigate whether miR-607 is useful as a prognostic biomarker that could provide useful information to existing prognostic factors. Univariate Cox regression analysis showed that clinicopathological factors including clinical stage (RR: 3.758, 95% CI: 1.566–7.383, *P* < 0.001), lymphatic metastasis (RR: 2.565, 95% CI: 1.368–4.893, *P*=0.005), liver metastasis (RR: 2.802, 95% CI: 1.251–4.943, *P*=0.001), perineural invasion (RR: 3.692, 95% CI: 1.702–6.659, *P* < 0.001), and miR-607 level (RR: 0.365, 95% CI: 0.169–0.790, *P*=0.011) were significantly associated with OS (Table 2). Also, multivariate Cox regression analysis was performed and results were shown in Table 3. We found that miR-607 level (RR: 0.449, 95% CI: 0.253–0.861, *P*=0.036) was an independent biomarker for the predicting of OS of PDAC patients.

### 3.5. Overexpression of miR-607 Inhibits PDAC Cell Migration and Invasion by Decreasing EMT Ability

**Figure 4:**
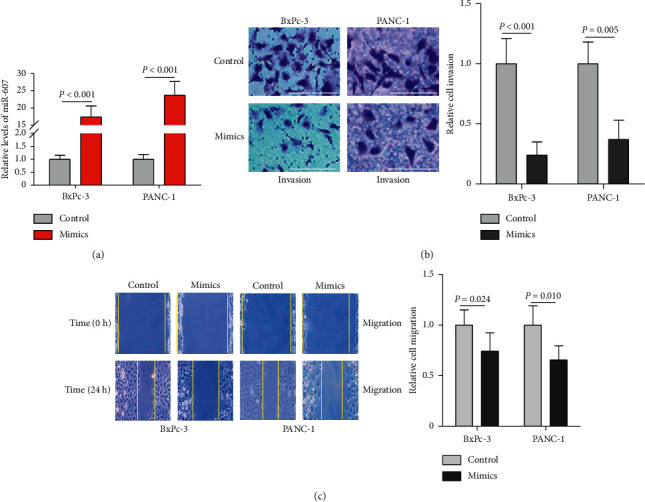
The effect of miR-607 on cell migration and invasion of PDAC cells. (a) Expression levels of miR-607 were detected in BxPc-3 and PANC-1 cells by qRT-PCR after transfection with miR-607 mimics and mimics control. (b) Representative results of cell invasion of PDAC cells by Transwell invasion assay at 24 h after cell transfection. Scale bar: 50 *μ*m. (c) Cell migration ability was determined by a wound-healing assay over 24 h after cell transfection. Results were expressed as mean ± SD for three replicate determination.

**Figure 5:**
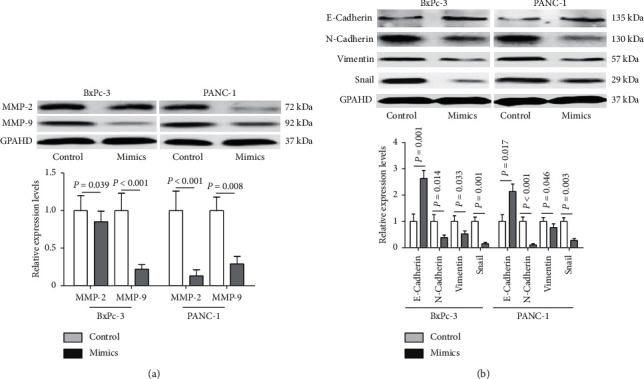
The impact of miR-607 on the EMT ability of PDAC cells. (a) Western blotting analysis assay confirmed the reduced expression of MMP-2 and MMP-9 (normalized to GAPDH) after miR-607 was overexpressed in BxPc-3 and PANC-1 cells. (b) Relative protein expression levels of EMT-related genes in PDAC cells after miR-607 overexpression were determined. Data were representative of at least three separate experiments.

Enhanced cell migration and invasion are key factors associated with cancer metastasis [22]. We, therefore, examined whether miR-607 affects these functions in PDAC cells metastasis. qRT-PCR confirmed that miR-607 expression was significantly increased in both BxPc-3 and PANC-1 cells 48 h after transfection of miR-607 mimics (Figure 4(a), *P* < 0.001). Subsequently, a Transwell invasion assay showed that upregulating the expression of miR-607 significantly inhibited invasiveness in PDAC cells (Figure 4(b), *P* < 0.01). Similarly, a wound-healing assay showed that overexpression of miR-607 significantly decreased cell migration ability (Figure 4(c), *P* < 0.05). In addition, the effect of miR-607 overexpression on the levels of MMP-2 and MMP-9 was determined. According to the Western blotting analysis results, the protein expression of MMP-2 and MMP-9 gene was reduced in the miR-607 mimics group compared with the mimics control group (Figure 5(a), *P* < 0.05).

Since EMT is closely related to cell migration and invasion [23], we examined whether the overexpression of miR-607 affects the protein expression of EMT-related genes. Western blotting analysis revealed that enforced expression of miR-607 in PDAC cells was associated with upregulation of epithelial marker E-cadherin and downregulation of mesenchymal markers including N-cadherin, Vimentin, and Snail (Figure 5(b), *P* < 0.05).

## 4. Discussion

Circulating miRNAs are considered stable molecular in the serum of the human body, which represent potential biomarkers for cancer diagnosis [24]. Also, profiling circulating miRNAs expression differences can help to make further staging and classification of cancer [25]. Moreover, several studies have reported that a combination of some circulating miRNA expressions by using bioinformatics methods may produce a more accurate prognosis for pancreatic cancer [26]. For example, it was reported that serum miR-499a-5p is a new, noninvasive biomarker, and the combination of miR-499a-5p and CA19-9 can improve the diagnostic sensitivity of pancreatic cancer [27]. A previous study showed that serum miR-629 is a novel molecular biomarker for diagnosis and the prognosis of pancreatic cancer [28]. In addition, serum miR-1290 and miR-1246 are promising biomarkers for pancreatic cancer diagnosis, and the two miRNAs' combined detection of CA19-9 could improve the diagnostic accuracy [29]. In this study, we investigated the clinical significance of miR-607 in PDAC tissues and serum samples, and the results showed that low serum miR-607 level may serve as a potential diagnostic and prognostic biomarker in PDAC patients. In a functional study, we found that miR-607 inhibits PDAC cell migration and invasion by decreasing EMT ability.

Although thousands of miRNAs have previously been identified in PDAC, investigation of their clinical values is relatively insufficient. Located at human chromosome 10q24.1, miR-607 has been confirmed to play a crucial role in a variety of pathological processes [30]. Up to date, there are no studies on the diagnostic and prognostic value of miR-607 in PDAC. Here, we observed that miR-607 levels were decreased in tumor tissues and serum samples from PDAC patients and PDAC cell lines. After analysis of the clinical significance and diagnostic value of miR-607, we found that miR-607 expression was firmly related to the lymphatic metastasis, liver metastasis, and perineural invasion of PDAC patients, raising the possibility that miR-607 is related to PDAC metastasis. The CEA and CA19-9 are two classic biomarkers of the diagnosis of PDAC [31]. In the current study, ROC curve analysis revealed that the miR-607 level has better potential for discriminating PDAC patients from healthy controls than CA19-9 and CEA levels. Notably, the diagnostic value of the combination of miR-607, CA19-9, and CEA was better than that alone, highlighting that miR-607 may be a potential biomarker for the diagnosis of PDAC.

In this study, the expression of serum miR-607 in PDAC patients before and after surgery was also analyzed, and its expression level showed an upward trend at 3, 6, and 12 months after surgery. Although the actual reasons were currently unclear, this cause might be due to the reduction of tumors and changes in the microenvironment. The increased expression of serum miR-607 in PDAC patients after surgery prompted us to identify whether miR-607 acts as a prognostic biomarker. Moreover, survival analysis of PDAC patients revealed that low serum miR-607 level is associated with poor OS and PFS. Lymphatic metastasis and perineural invasion are the strongest indicators of short OS in PDAC patients [32, 33]. Consistently, our studies showed that clinical stage, lymphatic metastasis, liver metastasis, perineural invasion, and miR-607 level were significantly associated with OS. Notably, the multivariate Cox proportional hazards model proved that low miR-607 level was an independent prognostic biomarker for predicting the poor OS of PDAC patients. These observations indicated that serum miR-607 may be a predictor of prognosis in PDAC. A limitation to our study was the relatively small number of clinical samples. Therefore, these conclusions must be treated with caution, and further studies with more clinical samples are warranted.

In previous studies, miRNA was shown to have anticancer function in cancer [10–12]. More recently, DCLK1, a putative marker of intestinal and pancreatic stem cells, plays a key regulatory role in pancreatic tumorigenesis through microRNA-dependent mechanisms and their downstream protumorigenic pathways [34]. A similar situation is observed with regard to miR-29b, which has been described to suppress tumor vascularization in pancreatic cancer, as well as tumor cell proliferation, invasion, and migration via negatively regulating VEGF-A expression [35]. It has been reported that miR-607 inhibits PC3 cell proliferation, colony formation, migration, and invasion by decreasing BLM RecQ like helicase gene expression [36]. Additionally, the suppressive effect of miR-607 on the proliferation and apoptosis in chronic lymphocytic leukemia by targeting frizzled class receptor 3 has been confirmed [19]. Thus, we performed wound-healing and Transwell invasion assays to investigate the migration and invasion of PDAC cells by transfection of miR-607 mimics in vitro.

Importantly, our data showed that overexpression of miR-607 decreased cell migration and invasion abilities of BxPc-3 and PANC-1 cells. It was reported that MMP-2 and MMP-9 play key roles in migration and invasion for PDAC cells [37, 38]. According to the Western blotting analysis results, we found that the protein expression of MMP-2 and MMP-9 genes was reduced in PDAC cells by miR-607 overexpression. During EMT, epithelial cells undergo profound phenotypic changes by loss of cell adhesion and cell polarity, but acquisition of cell migratory and invasive abilities, in which the downregulation of E-cadherin is balanced by upregulation of N-cadherin [39, 40]. Our study found that forced expression of miR-607 led to higher protein expression of E-cadherin and lower protein expression of N-cadherin, Vimentin, and Snail, which was consistent with previous reports. These results demonstrated that miR-607 inhibits PDAC cells metastasis through decreasing EMT ability. Further research is needed to focus on in vivo study to confirm the findings in our study.

It has been suggested that one miRNA can target ∼200 mRNAs, which may be responsible for multiple different proteins [41]. With the help of bioinformatics prediction (TargetScan, Pictar, and miRDB), we found that several genes, including PIK3CA, SCAI, HDAC9, FOXF1, GFPT1, USP31, MAP3K2, and SRSF2, had putative target sites of miR-607 in their 3′-UTRs. Previous studies have shown that some of these genes are closely related to the progression of PDAC [42–44]. However, which are the exact target genes of miR-607? What are the specific roles of these target genes in PDAC? Which cancer-related signaling pathway is miR-607 involved in? These questions are the purpose of further research.

In conclusion, we have identified that miR-607 is downregulated in tissues and serum samples of PDAC patients, and the low serum miR-607 level may serve as a potential diagnostic and prognostic biomarker in PDAC patients. We also confirmed that miR-607 inhibits PDAC cells metastasis via suppressing EMT. However, the mechanism underlying miR-607 implicated in the metastasis of PDAC is very complicated. Further investigation will be necessary to determine the exact mechanism of miR-607 regulating PDAC metastasis.

## Figures and Tables

**Table 1:** Relation between miR-607 expression and clinicopathological characteristics of PDAC patients.

Variables	Number	miR-607 expression		*P*
		Low (*n* = 25, %)	High (*n* = 25, %)	
Age (years)				0.087
<60	22	8 (16%)	14 (28%)	
≥60	28	17 (34%)	11 (22%)	

Gender				0.083
Male	30	12 (24%)	18 (36%)	
Female	20	13 (26%)	7 (14%)	

Tumor size (cm)				0.355
<3	15	9 (18%)	6 (12%)	
≥3	35	16 (32%)	19 (38%)	

Tumor differentiation				0.771
Well - moderate	19	10 (20%)	9 (18%)	
Poor	31	15 (30%)	16 (32%)	

Clinical stage				0.136
I-II	17	6 (12%)	11 (22%)	
III-IV	33	19 (38%)	14 (28%)	

T Stage				0.152
T1-T2	21	13 (26%)	8 (16%)	
T3-T4	29	12 (24%)	17 (34%)	

Lymphatic metastasis				0.002
Negative	14	2 (4%)	12 (24%)	
Positive	36	23 (46%)	13 (26%)	

Perineural invasion				0.004
Negative	22	6 (12%)	16 (32%)	
Positive	28	19 (38%)	9 (18%)	

Liver metastasis				0.018
Negative	45	20 (40%)	25 (50%)	
Positive	5	5 (10%)	0 (0%)	

CEA level (ng/mL)				0.774
<5	21	10 (20%)	11 (22%)	
≥5	29	15 (30%)	14 (28%)	

CA19-9 level (U/mL)				0.333
<35	13	8 (16%)	5 (10%)	
≥35	37	17 (34%)	20 (40%)	

PDAC: pancreatic ductal adenocarcinoma.

**Table 2:** Univariate Cox regression analysis of different prognostic factors for OS in PDAC patients.

Features	Univariate analysis		
	RR	95% CI	*P* value
Age (≥60 years vs. < 60 years)	1.132	0.706–1.894	0.442
Gender (male vs. Female)	0.763	0.488–1.268	0.715
Tumor size (≥3 cm vs. < 3 cm)	1.585	0.896–2.727	0.315
Differentiation (poor vs. Well - moderate)	1.749	0.950–3.419	0.062
Clinical stage (III-IV vs. I-II)	3.758	1.566–7.383	<0.001
T Stage (T3-T4 vs. T1-T2)	1.683	0.775–2.849	0.174
Lymphatic metastasis (positive vs. Negative)	2.565	1.368–4.893	0.005
Perineural invasion (positive vs. Negative)	3.692	1.702–6.659	<0.001
Liver metastasis (positive vs. Negative)	2.802	1.251–4.943	0.001
CEA level (≥5 ng/mL vs. < 5 ng/mL)	0.581	0.362–1.409	0.694
CA19-9 level (≥35 U/mL vs. < 35 U/mL)	1.601	0.718–2.972	0.072
miR-607 level (high vs. low)	0.365	0.169–0.790	0.011

OS: overall survival, RR: risk ratio, CI: confidence interval.

**Table 3:** Multivariate Cox regression analysis of different prognostic factors for OS in PDAC patients.

Features	Multivariate analysis		
	RR	95% CI	*P* Value
Age (≥60 years vs. < 60 years)	—	—	—
Gender (male vs. Female)	—	—	—
Tumor size (≥3 cm vs. < 3 cm)	—	—	—
Differentiation (poor vs. Well - moderate)	—	—	—
Clinical stage (III-IV vs. I-II)	2.136	0.932–3.510	0.064
T Stage (T3-T4 vs. T1-T2)	—	—	—
Lymphatic metastasis (positive vs. Negative)	1.472	0.733–2.659	0.218
Perineural invasion (positive vs. Negative)	1.755	0.619–3.021	0.077
Liver metastasis (positive vs. Negative)	1.246	0.402–2.115	0.430
CEA level (≥5 ng/mL vs. <5 ng/mL)	—	—	—
CA19-9 level (≥35 U/mL vs. <35 U/mL)	—	—	—
miR-607 level (high vs. low)	0.449	0.253–0.861	0.036

## Data Availability

The data used to support the findings of this study are available from the corresponding author upon request.
